# Root Resurgence: An Unexpected Twist After Natal Teeth Extractions

**DOI:** 10.7759/cureus.65907

**Published:** 2024-07-31

**Authors:** Kugendran V Rajendran, Fadzlinda Baharin, Habibah Md Said

**Affiliations:** 1 Paediatric Dentistry Unit, School of Dental Sciences, Universiti Sains Malaysia (USM), Kota Bharu, MYS; 2 Department of Paediatric Dentistry, Raja Perempuan Zainab II Hospital, Kota Bharu, MYS

**Keywords:** natal teeth, neonatal teeth, extraction, curettage, premature eruption

## Abstract

The presence of natal and neonatal teeth is a rare anomaly that can lead to various complications. This case report aims to highlight the potential delayed sequelae that can arise following the extraction of natal teeth. A boy aged three years and two months was referred for pain and an abscess on his lower anterior teeth. He had two natal teeth at birth, which were extracted on the second day of life due to excessive mobility and profound discomfort to the mother during breastfeeding. Surprisingly, he presented with pain in the same area where the teeth were extracted previously. Upon examination, residual root-like structures were observed at the sites of teeth 71 and 81, with an abscess noted on tooth 81. The radiographic assessment confirmed the presence of residual roots, prompting the extraction. History, clinical, radiographic, and histopathological evaluations strongly validate the diagnosis of residual natal teeth. Gentle curettage of the socket should be performed whenever possible following the extraction of natal teeth to prevent complications. Although residual tooth formation is uncommon, periodic follow-up is important to monitor for any potential problem and observe adjacent teeth eruption.

## Introduction

The first primary teeth typically emerge in the oral cavity as the mandibular central incisors, commonly appearing around the age of six months. However, in rare instances, teeth may even be present at birth or emerge shortly after birth. To be precise, natal teeth are those present at birth while neonatal teeth are those that erupt within the first month of life or the first 30 days of life [1]. These terms were first coined by Massler and Savara in 1950.

Multiple studies have verified a greater occurrence of natal teeth in comparison to neonatal teeth with a ratio of 3:1 [2,3]. This observation is further supported by the global prevalence estimates, which indicate approximately one in 289 newborns for natal teeth and one in 2,212 for neonatal teeth [4]. Natal teeth predominantly manifest in the mandibular central incisor region, accounting for approximately 85% of cases, followed by the maxillary incisor region at 11%. The mandibular canine and molar region contribute to 3% of occurrences, while the maxillary canine and molar region exhibit the lowest incidence at 1%. This distribution pattern coincides with the typical sequence of eruption for primary deciduous teeth [2,5]. The majority of studies have indicated a higher prevalence of this rare phenomenon in females, whereas some studies have reported no gender predilection [6]. Authors have indicated that natal and neonatal teeth are usually early eruptions from the normal set of primary teeth, 90-99%, with only 1-10% being supernumerary [7].

The exact cause of this condition remains uncertain [6]. However, potential contributing factors such as infection, malnutrition, hormonal stimulation, and the superficial positioning of the tooth germ in the infant have been suggested [1]. Meanwhile, some authors have proposed the potential inheritance of this condition through a familial autosomal dominant pattern [3]. The treatment of natal teeth may involve smoothing sharp edges, providing restoration coverage, or considering extraction, depending on the complications they cause [3]. This case report aims to underscore the potential delayed complications resulting from incomplete removal of natal teeth, including their follicles, despite the procedure initially perceived as straightforward.

## Case presentation

A Malay boy aged three years and two months with well-controlled bronchial asthma and recurrent bilateral congenital talipes equinovarus was referred to the Paediatric Dentistry Unit due to pain and a localized abscess on his lower anterior teeth. Given the rarity of an abscess on the mandibular incisor, further history was obtained. According to the mother, the patient was born full term via spontaneous vaginal delivery without complications, weighing approximately 2800 g at birth. Both parents had unremarkable medical histories. However, his younger brother had a similar history of a natal tooth in the 81 region, which was extracted and had an uneventful recovery. The mother noted the presence of two teeth on the lower anterior jaw at birth. Concerns about excessive tooth mobility and profound discomfort during breastfeeding heightened the mother's fear of pulmonary aspiration, prompting a referral to the dentist.

The mother asserted that she was informed these lower anterior erupted teeth could be supernumerary or possibly part of the primary dentition, as she preferred not to pursue a radiographic investigation for confirmation. Subsequently, on the second day of life, the patient underwent extraction of these teeth under topical anaesthesia and the mother resumed breastfeeding immediately. The extraction sites healed spontaneously without complications such as bleeding or infection. The mother stated that she observed the normal eruption of all other primary teeth and did not seek treatment for the missing lower anterior teeth, understanding the extracted teeth were indeed part of the primary dentition.

**Figure 1:**
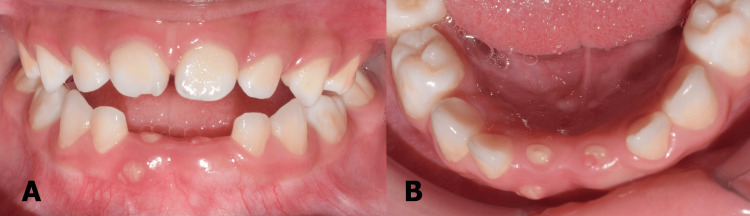
(A) Labial view (B) Lower occlusal view of residual roots of 71 and 81 with abscess on tooth 81.

Extraoral examination revealed no abnormalities while the intraoral examination was dominated by the spacing of clinically missing teeth 71 and 81. A residual root-like immobile structure was observed in the area of 71 and 81 with a labial localized abscess on the latter (Figure 1).

**Figure 2:**
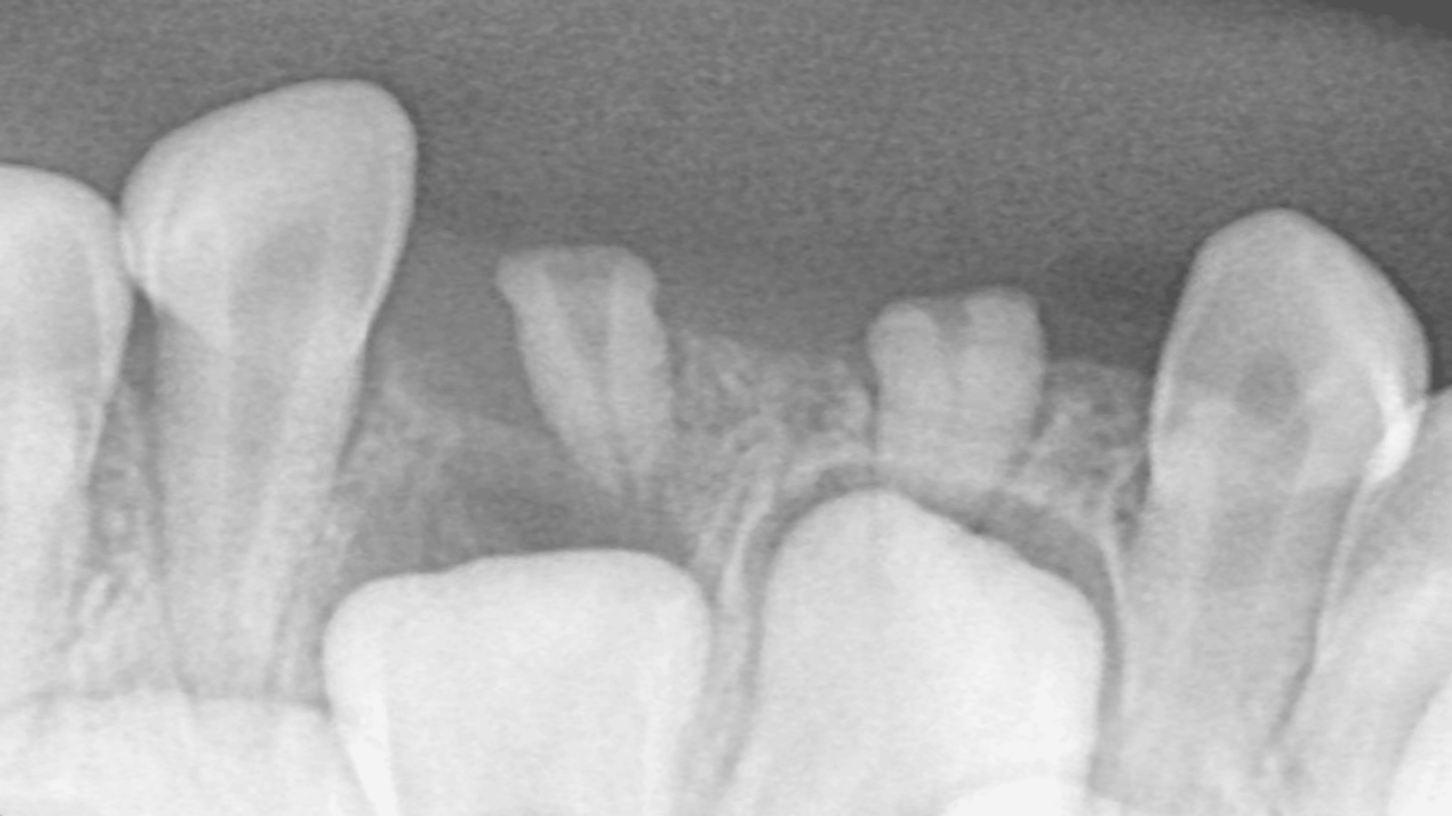
Periapical radiograph showing residual roots of teeth 71 and 81 with their respective successors.

The remaining primary teeth were all erupted normally, showing no signs of caries, and exhibiting good oral hygiene. A periapical radiograph was then taken to confirm whether the calcified structures were residual roots of the natal teeth or bony spicules. The radiograph revealed two small root-like structures with a thin pulp space. Moreover, the presence of the permanent successor strongly suggests that the previously extracted natal tooth was part of the primary dentition and not supernumeraries (Figure 2).

This history, along with clinical and radiographic investigations, validated that this is a case of residual natal tooth. The patient was scheduled for left Achilles tendon lengthening and posterior capsular release under general anaesthesia by the orthopaedic team. Taking advantage of the general anaesthesia session, we opted to perform the extraction of the retained roots concurrently, avoiding the need for a separate chairside extraction procedure. This approach was chosen to accommodate the patient's existing anxiety and minimize discomfort during the dental procedure.

**Figure 3:**
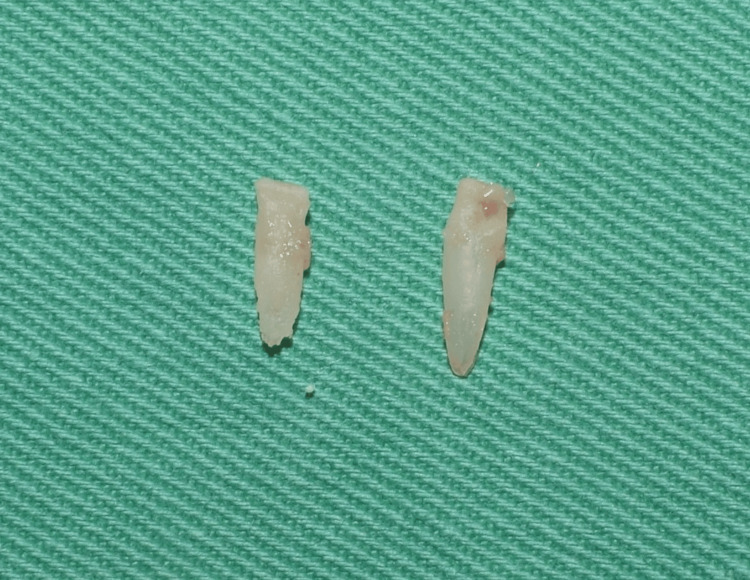
Extracted roots of teeth 71 and 81.

During the extraction, two small roots of teeth 71 and 81 were retrieved intact (Figure 3).

**Figure 4:**
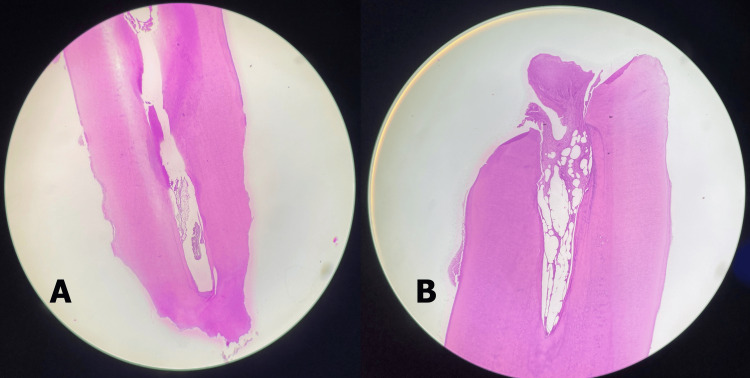
(A) This section shows dental hard tissue comprising of dentine with peripheral rim of cementum, exhibiting internal and external resorption with devoid of vital pulp tissue. (B) This section shows dental hard tissue comprising dentine with peripheral rim of cementum. The exposed pulp cavity shows fibrous tissue with dense inflammatory cell infiltrate within the exposed pulp (Hematoxylin and eosin staining: original magnification, 50x).

These fragments were sent for histopathological examination, which confirmed the presence of dental hard tissue consisting of dentine with a peripheral rim of cementum, exhibiting signs of external root resorption (Figure 4).

**Figure 5:**
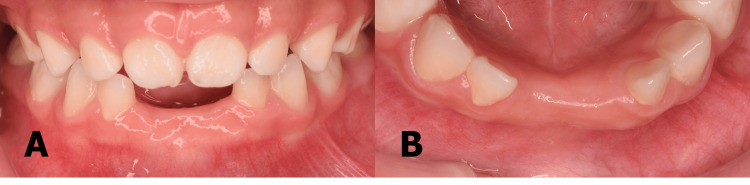
(A) Labial view (B) Lower occlusal view of healed extraction sockets of 71, 81, and resolved abscess.

The patient underwent a review appointment one month after the extraction, during which no active complaints were reported. The extraction site had healed, and the abscess had resolved (Figure 5). 

## Discussion

There are various complications associated with natal or neonatal teeth and symptomatic conditions are often the primary reason for referral. One of the complications of prematurely erupted teeth is the risk of pulmonary aspiration due to excessive mobility if dislodged during breastfeeding [7]. Additionally, discomfort and difficulty experienced by the mother due to nipple pain during breastfeeding add to the challenges of feeding [3]. Furthermore, traumatic ulcers on the ventral surface of the tongue, which is affected in 60% of cases [8], and other oral mucosa, known as Riga-Fede disease, resulting from repeated forward and backward movement of the tongue against the teeth, is also one of the contributing factors for the urgent referral [9,10]. This condition impedes the infant's ability to suckle and feed, increasing the risk of nutritional deficiencies and dehydration [6].

Depending on the clinical implication various treatment options have been discussed in the literature. In the cases of Riga-Fede, some clinicians prefer conservative approaches to maintain the neonatal tooth by smoothing or rounding the sharp incisal edge with an abrasive instrument, while others suggest covering the incisal edge with light-curable composites [6-8]. However, these teeth are usually hypomineralized and have a limited enamel surface for resin bonding. Additionally, challenges such as difficult access, moisture control, and enamel etching can make achieving adequate resin retention questionable. Some even recommended the use of glass ionomer cement as an alternative to resin-based material [9]. If the restoration fails, there is a risk that it could be swallowed or inhaled, potentially making the situation more detrimental despite the initial intention to alleviate symptoms.

Based on the available literature, in most cases, extraction was the treatment of choice, when there was excessive tooth mobility and unresolved ulceration [3]. Although the extraction of a natal tooth usually doesn't present significant difficulties, it must be executed with utmost care and attention. Complications after extraction are exceedingly rare, although some authors have documented instances of natal or neonatal tooth recurrence or continued growth of the radicular mass. In a study by Rahul et al., out of 44 children, four (9.1%) developed tooth-like structures after the initial extraction of premature teeth. Two of these children experienced infections that led to alveolar abscesses, necessitating the extraction of residual tooth-like structures [11], similar to our case. In another study by Samuel et al. involving 52 natal and neonatal teeth, a residual natal tooth was observed in one case where routine curettage was not practiced [12]. Additionally, in a case report by Anton et al., an asymptomatic residual immobile tooth-like structure was discovered in the lower mandibular incisor region during a follow-up examination conducted two years post-extraction of the natal tooth [5]. Another case report described an atypical tooth formation with an abscess in the position of the mandibular right primary central incisor, which had been a natal tooth extracted at two weeks of age due to Riga-Fede disease [13]. A case reported by Dyment et al. involved a nine-month-old patient who presented with root-like hard tissues visible at the anterior mandibular alveolar ridge at the sites previously diagnosed as neonatal teeth. The neonatal tooth at position 71 had spontaneously exfoliated, and the one at position 81 had been extracted at 3 weeks of age without curettage [14]. Besides that, there was a case, reported by Sultan et al., of a pre-term baby who spontaneously exfoliated the neonatal tooth 71 at 40 weeks of life. At the subsequent three-month follow-up, a calcified tissue was noted in the socket of 71. No curettage could be performed as the tooth had exfoliated naturally [15]. Another study by Kim et al. reported the development of tooth-like structures following the extraction of neonatal teeth in eight cases they investigated over 47 years, from 1962 to 2009 [16].

Some authors have proposed that this occurs because, during the extraction of a natal tooth, only the external hard tissue is removed, leaving the internal part of the dental papilla. This is because the underdeveloped cells of the dental papilla and Hertwig's root sheath can easily detach from the calcified part of the tooth. This exposed part of the dental papilla, along with odontoblasts and remnants of the Hertwig epithelial root sheath, which remain within the socket, can retain their vitality. It is hypothesized that the mesenchymal stem cells present in the dental papilla undergo morpho-differentiation into various cell types, including osteoblasts, chondrocytes, adipocytes, endothelial cells, and odontoblasts, ultimately leading to the formation of dental structures [11,17,18]. Therefore, all these studies recommended curettage of the socket gently following the extraction to ensure that the underlying dental papilla and Hertwig's epithelial root sheath are carefully removed to prevent the continued development of tooth-like structures [7,14,15].

Premature loss of primary incisors typically does not necessitate space maintenance, as it generally has minimal effects on the developing dentition [19]. Although premature loss of primary incisors can lead to untoward outcomes such as eruption disturbances of the permanent successors, inclination of adjacent teeth, crowding, speech problems, and psychosocial consequences, these issues are more commonly associated with maxillary incisors rather than mandibular incisors, as reported in the literature [20]. In this case, since the patient's parents had no aesthetic concerns and considering his uncooperative nature, periodic follow-up was chosen over using an appliance.

## Conclusions

While residual tooth formation is uncommon following extraction, it is important to acknowledge the associated risks. Therefore, parents should be adequately informed and encouraged to ensure regular dental check-ups for their children. Even if a discharge is typical after natal/neonatal teeth extraction, considering placing such a patient under periodic review, perhaps annually, would be prudent. This allows timely monitoring for residual teeth, as well as the eruption of successors, ensuring optimal oral health.

## References

[1] Shivpuri A, Mitra R, Saxena V, Shivpuri A (2021). Natal and neonatal teeth: clinically relevant findings in a retrospective analysis. Med J Armed Forces India.

[2] Brummund D, Chang A, Michienzi J (2022). Pedunculated natal tooth: a case report. Cureus.

[3] Mhaske S, Yuwanati MB, Mhaske A, Ragavendra R, Kamath K, Saawarn S (2013). Natal and neonatal teeth: an overview of the literature. ISRN Pediatr.

[4] Vitali FC, Santos PS, Massignan C, Cardoso M, Maia LC, Paiva SM, Teixeira CD (2023). Worldwide prevalence of natal and neonatal teeth: systematic review and meta-analysis. J Am Dent Assoc.

[5] Anton E, Doroftei B, Grab D (2020). Natal and neonatal teeth: a case report and mecanistical perspective. Healthcare (Basel).

[6] Jamani NA, Ardini YD, Harun NA (2018). Neonatal tooth with Riga-Fide disease affecting breastfeeding: a case report. Int Breastfeed J.

[7] Malki GA, Al-Badawi EA, Dahlan MA (2015). Natal teeth: a case report and reappraisal. Case Rep Dent.

[8] Costacurta M, Maturo P, Docimo R (2012). Riga-Fede disease and neonatal teeth. Oral Implantol (Rome).

[9] Volpato LE, Simões CA, Simões F, Nespolo PA, Borges ÁH (2015). Riga-fede disease associated with natal teeth: two different approaches in the same case. Case Rep Dent.

[10] Mohan RP, Verma S, Gill N, Singh U (2014). Riga-Fede disease (Cardarelli’s aphthae): a report of nine cases. South Africa J Child Health.

[11] Rahul M, Kapur A, Goyal A (2018). Management of prematurely erupted teeth in newborns. BMJ Case Rep.

[12] Samuel SS, Ross BJ, Rebekah G, Koshy S (2018). Natal and neonatal teeth: a tertiary care experience. Contemp Clin Dent.

[13] Nedley MP, Stanley RT, Cohen DM (1995). Extraction of natal and neonatal teeth can leave odontogenic remnants. Pediatr Dent.

[14] Dyment H, Anderson R, Humphrey J, Chase I (2005). Residual neonatal teeth: a case report. J Can Dent Assoc.

[15] Sultan A, Kaur G, Antony TJ, Kumar SS (2019). Residual neonatal tooth in a pre-term infant: a case report and brief review. Int J Oral Health Dentistry.

[16] Kim SH, Cho YA, Nam OH, Kim MS, Choi SC, Lee HS (2016). Complication after extraction of natal teeth with continued growth of a dental papilla. Pediatr Dent.

[17] Ooshima T, Mihara J, Saito T, Sobue S (1986). Eruption of tooth-like structure following the exfoliation of natal tooth: report of case. ASDC J Dent Child.

[18] Tsubone H, Onishi T, Hayashibara T, Sobue S, Ooshima T (2002). Clinico-pathological aspects of a residual natal tooth: a case report. J Oral Pathol Med.

[19] Watt E, Ahmad A, Adamji R, Katsimpali A, Ashley P, Noar J (2018). Space maintainers in the primary and mixed dentition - a clinical guide. Br Dent J.

[20] Nadelman P, Magno MB, Pithon MM, Castro AC, Maia LC (2021). Does the premature loss of primary anterior teeth cause morphological, functional and psychosocial consequences?. Braz Oral Res.

